# Restraint stress induced gut dysmotility is diminished by a milk oligosaccharide (2′-fucosyllactose) *in vitro*

**DOI:** 10.1371/journal.pone.0215151

**Published:** 2019-04-24

**Authors:** Sohana Farhin, Annette Wong, Thilini Delungahawatta, Jessica Y. Amin, John Bienenstock, Rachael Buck, Wolfgang A. Kunze

**Affiliations:** 1 McMaster Brain-Body Institute, St. Joseph’s Healthcare, Hamilton, Ontario, Canada; 2 Department of Pathology and Molecular Medicine, McMaster University, Hamilton, Ontario, Canada; 3 Abbott Nutrition R&D, RP4-3/Dept 105600, Columbus, Ohio, United States of America; 4 Department of Psychiatry and Behavioural Neurosciences, McMaster University, Hamilton, Ontario, Canada; 5 Department of Biology, McMaster University, Hamilton, Ontario, Canada; University of California Los Angeles, UNITED STATES

## Abstract

**Background:**

Stress causes severe dysmotility in the mammalian gut. Almost all research done to date has concentrated on prevention of stress-induced altered gut motility but not on treatment. We had previously shown that intraluminal 2′FL could acutely moderate propulsive motility in isolated mouse colonic segments. Because 2′FL appeared to modulate enteric nervous system dependent motility, we wondered if the oligosaccharide could reverse the effects of prior restraint stress, ex vivo. We tested whether 2′FL could benefit the dysmotility of isolated jejunal and colonic segments from animals subjected to prior acute restraint stress.

**Methods:**

Jejunal and colonic segments were obtained from male Swiss Webster mice that were untreated or subjected to 1 hour of acute restraint stress. Segments were perfused with Krebs buffer and propagating contractile clusters (PCC) digitally video recorded. 2′FL or β-lactose were added to the perfusate at a concentration of 1 mg/ml. Spatiotemporal maps were constructed from paired before and after treatment recordings, each consisting of 20 min duration and PCC analyzed for frequency, velocity and amplitude.

**Key Results:**

Stress decreased propulsive motility in murine small intestine while increasing it in the colon. 2′FL in jejunum of previously stressed mice produced a 50% increase in PCC velocity (p = 0.0001), a 43% increase in frequency (p = 0.0002) and an insignificant decrease in peak amplitude. For stressed colon, 2′FL reduced the frequency by 23% (p = 0.017) and peak amplitude by 26% (p = 0.011), and was without effect on velocity. β-lactose had negligible or small treatment effects.

**Conclusions & Inferences:**

We show that the prebiotic 2′FL may have potential as a treatment for acute stress-induced gut dysmotility, ex vivo, and that, as is the case for certain beneficial microbes, the mechanism occurs in the gut, likely via action on the enteric nervous system.

## Introduction

Environmental stress is associated with disordered peristalsis and diarrhea [1]. Indeed, intestinal motility appears to be particularly sensitive to stress [2, 3]. These observations apply also to our everyday life, animal husbandry and experimental studies of animal behavior. In the experimental literature, prevention of stress effects on the gut has received considerable attention [4–11], whereas treatment of these (i.e. after stress) has received very little. There are few treatments available clinically for stress-induced gut dysmotility, but the one most used is loperamide, a μ-opioid receptor agonist, which inhibits peristalsis but does not restore normal oral to anal propulsive motility characterized by propagating contractile clusters (PCC) [12, 13]. We have reported that a neuroactive bacterium, *Lactobacillus rhamnosus* JB-1, could partially reverse in vitro the effects of prior acute restraint stress on murine gut dysmotility [14]. However, there are few additional substances with similar treatment effects.

While human milk oligosaccharides (HMOs) were originally only considered for their prebiotic effects on gut bacteria, a fucosylated HMO, 2′-fucosyllactose (2′FL), has acute regulatory effects on PCC in mouse colon taken from unstressed animals [15]. However, the effects of treatments on unstressed gut tissue do not predict their action on gut taken from previously stressed animals. For example, intraluminal JB-1 decreased PCC frequency in jejunal segments taken from unstressed mice [16], but increased frequency in jejunum taken from stressed mice [14]. Thus, while 2′FL alters propulsive peristalsis in unstressed colon 2′FL, it is unknown whether it has potential to reverse stress induced dysmotility.

An acute restraint model of stress was chosen because the stressor is easily applied, and because the effects on gut motility are highly reproducible and have been comprehensively established [2, 3, 17]. Specifically, acute restraint stress decreases propulsive motility in the murine small intestine while increasing it in the colon [14, 17]. We tested the effects of intraluminal 2′FL application on in vitro motility of gut segments taken from previously stressed mice; these results were compared to the effects of intraluminal β-lactose on similar segments. 2’-FL was selected because it is the most abundant HMO. β-lactose was used as a control for the effects of 2′FL because it has been used as a control for 2′FL in a significant number of publications including our original one on motility [15]. The reason that β-lactose has been so used is that the test oligosaccharide (2′FL) is fucosylated lactose. In fact it is likely that human milk oligosaccharides generally are synthesised by enzyme action on β-lactose [18].

Intestinal propulsive reflexes occur in intestinal segments placed in an ex vivo organ bath and they also occur in vivo after all connections between the intestine and the central nervous system have been severed [19]. Thus, because intestinal motility is almost entirely controlled by the intramural enteric nervous system it might be predicted that the effects of stress and 2′FL observed in vitro would be replicated with in vivo studies. To test this we have used an in vivo model were mice were restraint stressed or not to determine the effects of stress on faecal pellet production, in addition a separate cohort of stressed mice were fed 2′FL immediately after the stress period was completed to determine the effects of 2′FL on pellet production.

## Materials and methods

### Animals

Adult male Swiss Webster mice (6–8 w) were purchased from Charles River Laboratories (Wilmington, MA, USA) and allowed to acclimatize for 1 week, 3-5/cage with 12 h light/dark schedule and food and water ad libitum. Mice were fed a standard rodent low fat diet: Teklad 8640, Teklad diets (formerly Harlan), Madison, WI.

Mice were either stressed by placement in a wire mesh restraint device for 1 h or kept in their cage for the same duration. They were then killed by cervical dislocation [14]. All experiments were conducted as approved by the McMaster University Animal Research Ethics Board (permit 08-08-35).

### Gut motility recordings

All gut motility recordings of the jejunum and colon were conducted according to published methods [14, 16]. 4 cm segments of proximal jejunum and colon were gently flushed out with Krebs saline, oral and anal ends cannulated with silicone tubing and attached orally to Mariotte’s flasks [20] allowing perfusion with oxygenated Krebs buffer or buffer to which either 2′FL or β-lactose was added. Hydrostatic pressures within the segment’s lumen at the oral end were 2 hPa for the jejunum and 2–3 hPa for the colon with the outflow elevated above the inflow level by 0.2 cm. The resulting intraluminal fluid pressure evoked repeated PCC running in the oral to anal direction [14, 21] and >90% spanned more than half of the segment’s length. Shorter PCC were not analysed further [21].

**Fig 1:**
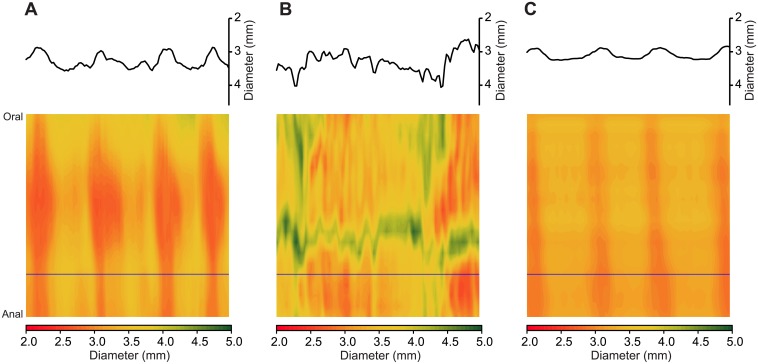
Spatiotemporal Dmaps showing effect of 2′FL on stressed jejunal segment in vitro. Spatiotemporal diameter maps for which the top to bottom axis gives the oral-anal distance (4 cm) and time is represented from left to right (300 s). The gut diameter at each locus is scaled to a red-green heat map with red representing contraction and green relaxation. A diameter vs time plot for a single level along the oral-anal axis (indicated by horizontal line) is inserted above each spatiotemporal map. A) Unstressed jejunum. B) Jejunum taken from previously restraint-stressed animal showing reduction in coordinated oral to anal PCCs. C) Diameter map made from the same *ex vivo* segment as for B but starting 5 min after 2′FL was added to the lumen showing increased occurrence of regular coordinated PCCs.

Motility was video-recorded on a JVC camcorder placed 10 cm above the gut segment and stored on computer. Post hoc video processing and construction of spatiotemporal diameter maps (Dmaps) were performed using “imageJ” software (https://imagej.nih.gov/ij/), and imageJ plug-ins: both in-house [16] and "d maple" (http://scepticalphysiologist.com/code/code.html). Dmaps are shown as image heat maps with the oral-anal direction running top to bottom and time running from left to right. For each pixel making up the Dmap, the intestine’s diameter is color coded with red denoting contraction and green relaxation (for example Fig 1). PCC are revealed in Dmaps as broad red bands [14, 22] moving in the oral to anal direction and are dependent on enteric nervous system (ENS) activity because they are abolished by the Na channel blocker tetrodotoxin [16, 23, 24].

PCC velocities were measured from the slope of the red bands (distance/time) and frequencies were measured from intervals between successive bands. Amplitude was measured as the differences in diameter between baseline and contraction peak. Changes to PCC measurements between control and treatment experiments indicate neuronally-dependent modulation of contractile peristaltic wave propagation.

### Experimental Design

Two types of experiments were performed ex vivo; jejunum or colon segments taken from unstressed mice were tested, *in vitro*, for effects on motility of intraluminal saccharides; or mice were stressed and *in vitro* segments tested with the saccharides. For each mouse, either a colon segment or a jejunal segment (but not both) were tested since only one segment could fit into the recording bath at a time, and testing jejunum and colon sequentially would have varied the interval between the stress period and the onset of motility recording. Sixteen unstressed jejunal segments were tested. For stressed jejunum, 21 segments were tested with 2′FL, 23 with β-lactose. For stressed colon, 33 segments were tested for 2′FL and 12 for β-lactose. Mice were subjected to restraint stress for 60 min and sacrificed by cervical dislocation 10 min later. Altogether, the total time elapsing between the end of stress and the beginning of video recording ranged from 45–60 min. Recordings were initially made while Krebs buffer was perfused luminally and compared to those made when the perfusate was switched to Krebs containing added 2′FL or β-lactose. The actual perfusion of intestinal segments with 2′FL or β-lactose each consisted of a 20 min period. Control recordings in stressed mice perfused with Krebs buffer alone were also for a period of 20 min.

2′FL was a gift from Abbott Nutrition, Columbus, Ohio and added to the luminal perfusing solution at 1 mg/ml of Krebs buffer. β-lactose was obtained from Sigma-Aldrich Co., St. Louis, MO, dissolved in Krebs buffer at 1 mg/ml. We chose 1 mg/ml as the test does because previous experiments have shown that as little as 0.5 mg/ml of 2’FL significantly reduces mouse colon PCC velocity and frequency and 1 mg/ml reduced PCC amplitude by at least 50% [15].

For in vivo studies of faecal pellet expulsion, mice were restraint stressed for 1 h, then each mouse was placed in a clean cage and gavaged with either 0.2 mL PBS or 2 mg 2′FL in 0.2 mL PBS. Then, after waiting 1 h, the pellets produced during next 1 h were collected to be counted and weighed.

### Statistics

Descriptive statistics are given in results tables as mean ± SD with number of gut segments in brackets. Treatment effects are given in the text as % mean differences and as probability of superiority (PS) based on the distribution of difference scores and standard deviations [25]. Where PS is the probability that in a randomly sampled pair of scores (one matched pair) the score from condition A will be greater than the score from condition B. We used the paired t test with a null hypothesis of no difference to compare sample group means before and after adding 2′FL, or β-lactose; sample sizes and p-values are given in results tables.

## Results

We verified that 1 hour acute restraint stress affected motility as we have previously reported [14, 22] (Table 1). Namely, stress decreased PCC velocity and frequency while increasing amplitude for jejunum, but increased PCC velocity and frequency with negligible effects on amplitude for colon.

**Table 1:** Effects of stress on PCC parameters.

	Unstressed	Stressed	P (t-test)
Jejunum			
Velocity (mm/s)	2.543 ± 0.081 (32)	1.457 ± 0.275 (44)	<.001
Frequency (Hz)	0.02667 ± 0.00003 (32)	0.01965 ± 0.00014 (46)	.001
Peak amplitude (mm)	0.506 ± 0.008 (32)	0.666 ± 0.037 (44)	<.001
Colon			
Velocity (mm/s)	0.752 ± 0.038 (14)	1.399 ± 0.509 (45)	<0.001
Frequency (Hz)	0.00601 ± 0 (14)	0.009 ± 0.00002 (45)	<0.001
Peak amplitude (mm)	0.615 ± 0.01 (14)	0.627 ± 0.087 (45)	0.82

Restraint stress decreased ex vivo propagated contractile complex (PCC) velocity and frequency for jejunal segments but increased peak amplitude. For colon, stress increased PCC velocity and frequency with negligible effects on amplitude. Values are mean ± SD (N).

### Jejunum

2′FL increased propulsive motility for stressed jejunal segments (Fig 1) whereas ß-lactose had negligible or small effects. The onset latencies for the motility increase after adding 2′FL ranged from 10 to 15 min, with similar onset latencies of action recorded for effects on the colon (see below).

**Fig 2:**
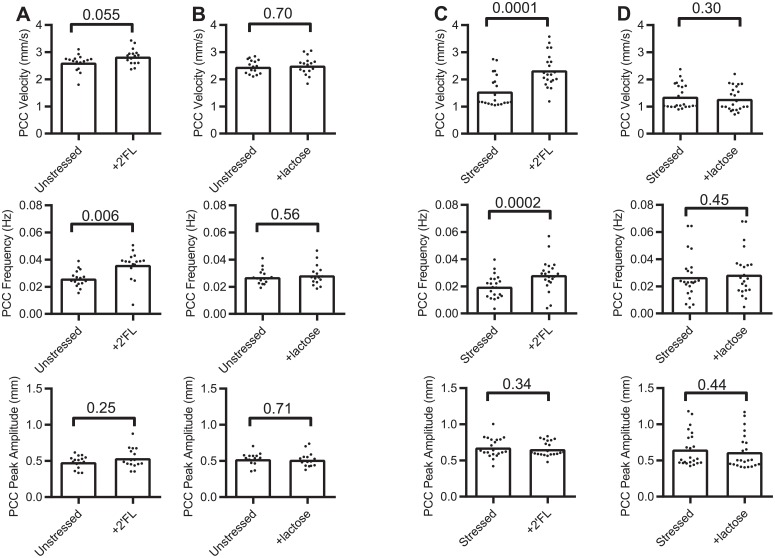
Dot plot graphs of the effects of 2′FL or ß-lactose on jejunal segments. Propagating contractile cluster (PCC) parameters before and after adding luminal saccharides measured in *ex vivo* segements. (A & B) unstressed mice, (C & D) stressed mice. Bars represent means.

For unstressed jejunum, and comparing Krebs + 2′FL vs Krebs only, PCC parameter sample means differences and related PS (in brackets) were: +8% (88%) for velocity, +39% (94%) for frequency and +11% (75%) for peak amplitude (Fig 2, Table 2). β-lactose effects on jejunum from unstressed mice for the same parameters were: velocity +2% (60%), frequency +5% (64%) and peak amplitude -2% (59%) (Fig 2, Table 2).

**Table 2:** Effects on PCC parameters of 2′FL or β-lactose on mouse jejunum.

Unstressed	Krebs	+2′FL	N, P (paired t test)
Velocity (mm/s)	2.62 ± 0.304	2.84 ± 0.298	16, 0.055
Frequency (Hz)	0.026 ± 0.078	0.036 ± 0.01	16, 0.006
Amplitude (mm)	0.485 ± 0.087	0.540 ± 0.139	16, 0.25
Unstressed	Krebs	+β-lactose	N, P (paired t test)
Velocity (mm/s)	2.466 ± 0.252	2.505 ± 0.333	16, 0.70
Frequency (Hz)	0.0272 ± 0.006	0.0285 ± 0.008	16, 0.56
Amplitude (mm)	0.527 ± 0.084	0.518 ± 0.097	16, 0.71
Stressed	Krebs	+2′FL	N, P (paired t test)
Velocity (mm/s)	1.559 ± 0.58	2.337 ± 0.613	21, 0.0001
Frequency (Hz)	0.018 ± 0.009	0.025 ± 0.012	21, 0.0002
Amplitude (mm)	0.680 ± 0.134	0.660 ± 0.104	21, 0.34
Stressed	Krebs	+β-lactose	N, P (paired t test)
Velocity (mm/s)	1.363 ± 0.459	1.283 ± 0.441	23, 0.30
Frequency (Hz)	0.027 ± 0.016	0.029 ± 0.018	23, 0.45
Amplitude (mm)	0.654 ± 0.237	0.617 ± 0.241	23, 0.44

For stressed jejunum, 2′FL changed PCC sample means by +50% (98%) for velocity and +42% (95%) and -3% (68%) for frequency and amplitude respectively (Figs 1 & 2, Table 2). Whereas, ß-lactose altered the same parameters by -6% (68%), +7% (63%) and -6% (65%) (Fig 2, Table 2).

### Colon

**Fig 3:**
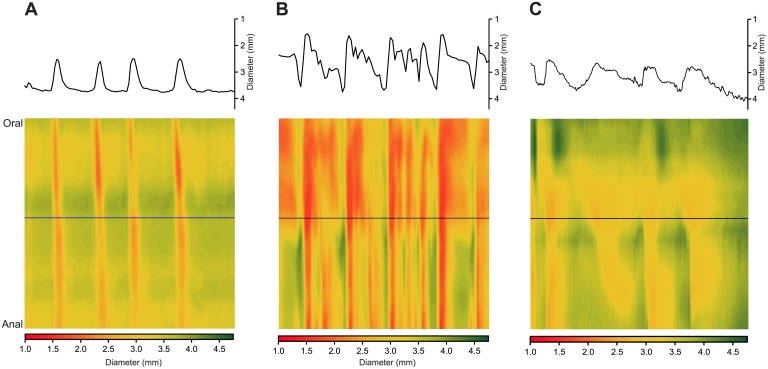
Spatiotemporal maps showing effect of 2′FL on stressed colon segment. Maps were constructed using the same parameters for time and distance as for Fig 1) Unstressed colon. B) Colon taken from previously restraint-stressed animal showing increased frequency of oral to anal PCCs. C) Diameter map made from the same ex vivo segment as for B but starting 5 min after 2′FL was added to the lumen showing reduction in the number of PCCs.

2′FL decreased propulsive motility for stressed colon segments (Fig 3); however, β-lactose had minor effects except for stressed colon PCC amplitude, which was increased.

**Fig 4:**
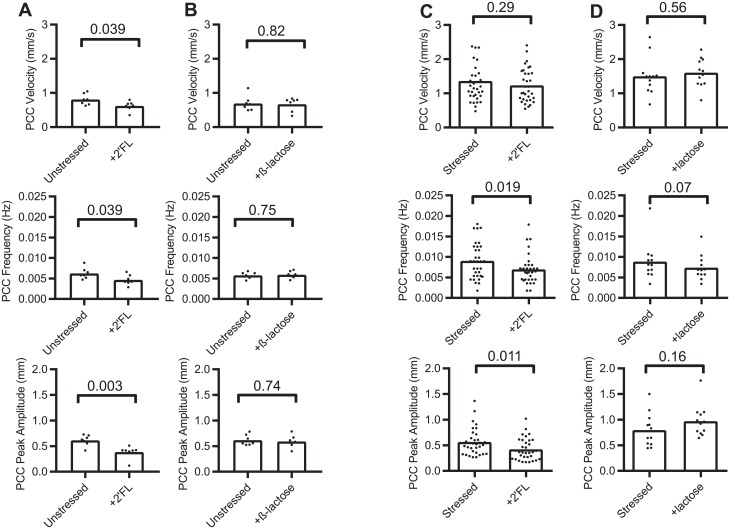
Dot plot graphs of the effects of 2′FL or ß-lactose on colon segments. Propagating contractile cluster (PCC) parameters before and after adding luminal saccharides measured in *ex vivo* segments. (A & B) unstressed mice, (C & D) stressed mice. Bars represent means.

For stressed colon, 2′FL altered velocity by -10% (63%), frequency by -23% (76%) and amplitude by -26% (78%). For unstressed colon, 2′FL altered sample velocity by -23% (99%), frequency by -26% (99%) and amplitude by -37% (99%). β-lactose altered velocity for unstressed colon by-3.5% (64%), frequency by +2% (69%) and amplitude by -4% (69%). For stressed colon β-lactose altered velocity by +7% (66%), frequency by +3% (54%) and amplitude by +22% (88%) (Fig 4, Table 3).

**Table 3:** Effects on PCC parameters of 2′FL or β-lactose on mouse colon.

Unstressed	Krebs	+2′FL	N, P (paired t test)
Velocity (mm/s)	0.808 ± 0.026	0.621 ± 0.019	7, 0.039
Frequency (Hz)	0.00625 ± 0.0007	0.00466 ± 0.0001	7, 0.039
Amplitude (mm)	0.614 ± 0.011	0.385 ± 0.015	7, 0.003
Unstressed	Krebs	+β-lactose	N, P (paired t test)
Velocity (mm/s)	0.696 ± 0.22	0.672 ± 0.199	7. 0.82
Frequency (Hz)	0.00577 ± 0.00076	0.00592 ± 0.00086	7, 0.75
Amplitude (mm)	0.617 ± 0.103	0.592 ± 0.12	7, 0.74
Stressed	Krebs	+2′FL	N, P (paired t test)
Velocity (mm/s)	1.362 ± 0.773	1.232 ± 0.494	33, 0.29
Frequency (Hz)	0.0091 ± 0.066	0.0070 ± 0.004	33, 0.019
Amplitude (mm)	0.565 ± 0.261	0.42 ± 0.213	33, 0.011
Stressed	Krebs	+β-lactose	N, P (paired t test)
Velocity (mm/s)	1.502 ± 0.534	1.603 ± 0.416	12, 0.56
Frequency (Hz)	0.0088 ± 0.005	0.0091 ± 0.003	12, 0.07
Amplitude (mm)	0.798 ± 0.324	0.971 ± 0.305	12, 0.16

### Myogenic contractions

**Fig 5:**
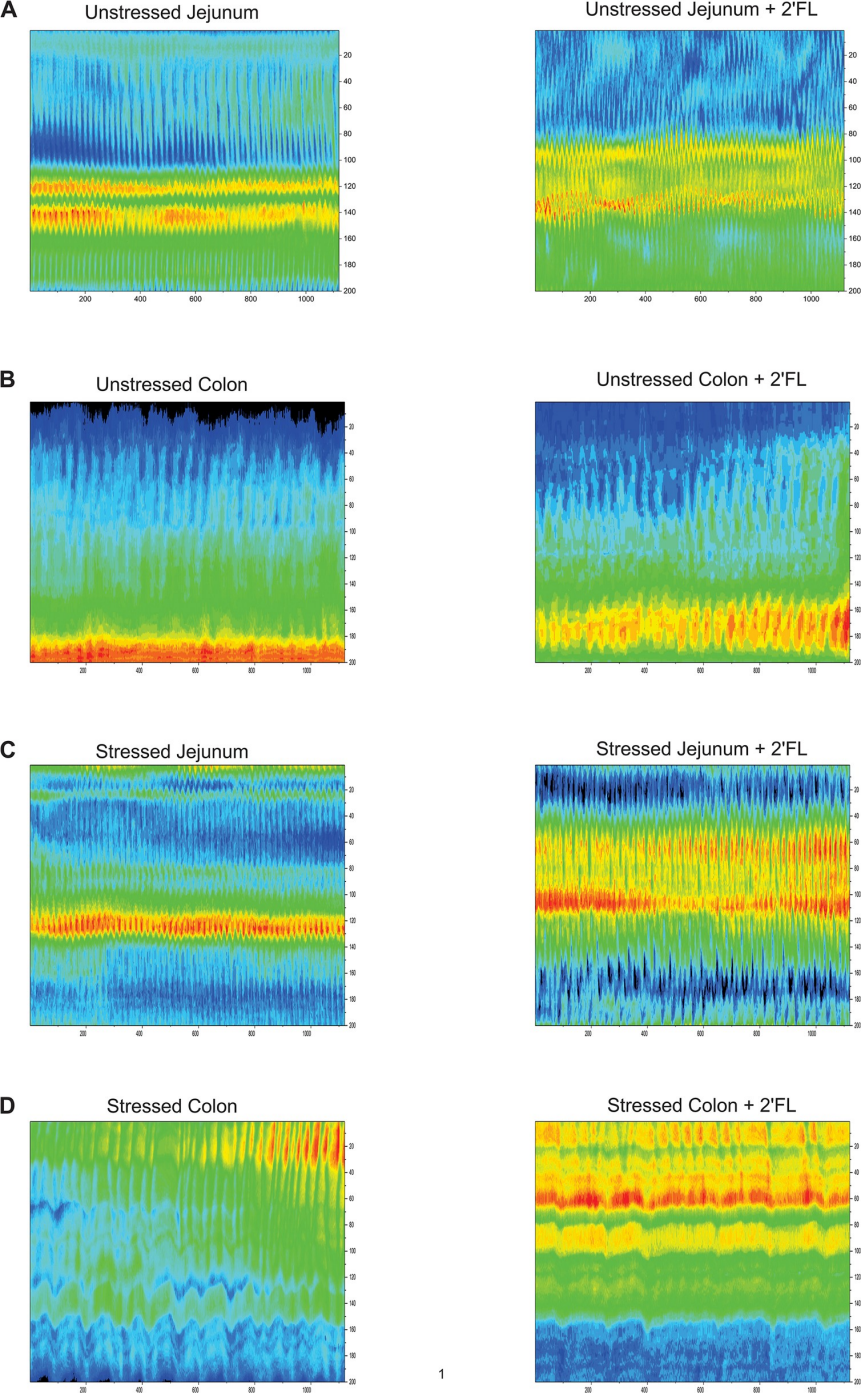
Spatiotemporal maps showing effects of 2′FL on myogenic contractions (ripples) after first adding tetrodotoxin (TTX) to the superfusate to abolish PCCs. Blue bands represent contractions and yellow-red bands relaxations of gut diameter. (A) Before (left panel) and after (right) panel adding 2′FL to the Krebs saline perfusing the lumen of jejunal segment taken from unstressed mouse. Before and after adding 2′FL to unstressed colon (B), unstressed jejunum (C) and stressed jejunum (D).

**Fig 6:**
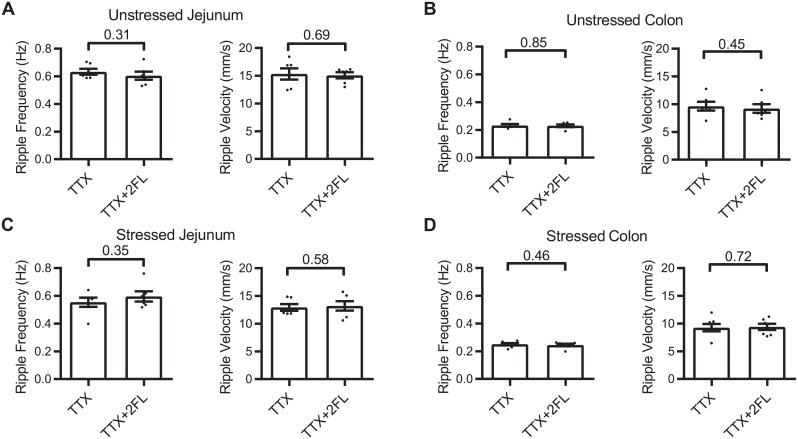
Dot plot graphs of the effects 2′FL on myogenic contractions (ripples) in the presence of TTX. 2′FL was without significant effect on myogenic contractions after neuronal activity was silenced by applying tetrodotoxin. Unstressed (A) and stressed (B) jejunum; (C) unstressed and (D) stressed colon.

To determine whether 2′FL induced alterations in PCC can be attributed to a direct action on contractions of the circle smooth muscle (ripples), we recorded motility after first blocking PCC by adding 0.3 μM tetrodotoxin (TTX) to the Krebs buffer superfusing the serosal surface of the gut segments (Fig 5). 2′FL appear to have no significant effects on ripple velocity or frequency (Fig 6) indicating that 2′FL alters PCC velocity and frequency via action on the enteric nervous system.

### Effects of 2′FL ingestion on restraint stress induced increase in faecal pellet production, in vivo

Restraint stress increased faecal pellet production and weight and this increase was significantly reduced by feeding 2′FL by oral gavage. Unstressed mice fed PBS produced 2.7 ± 0.7 (N = 6), stressed mice fed PBS produced 11.2 ± 1.7 (N = 6) and stressed mice fed 2′FL produced 4.8 ± 1.2 (N = 6) faecal pellets per hour. Pellet production for PBS fed unstressed vs PBS fed stressed mice differed statistically (p = 0.0005) and production for PBS fed stressed vs 2′FL fed stressed mice also differed statistically (p = 0.003) (Holm-Sidak’s multiple comparison tests). Faecal pellets from unstressed mice fed PBS weighed 0.080 ± 0.017, they weighed 0.235 ± 0.053 for stressed mice fed PBS, and 0.085 ± 0.018 g for stressed mice fed 2′FL. Pellet weight for PBS fed unstressed vs PBS fed stressed mice differed statistically (p = 0.014) and weight for PBS fed stressed vs 2′FL fed stressed mice also differed statistically (p = 0.014).

## Discussion

The present report demonstrates for the first time that a single oligosaccharide, 2′FL, rather than a live microbe [14] can have stress-reversing effects on intestinal motility. We have shown clear treatment effects of 2′FL relative to a control carbohydrate, β-lactose, on restraint stress-induced gut dysmotility. 2′FL largely reversed the slowed PCC velocity and frequency in jejunum, and increased frequency and peak amplitude in colon.

The *in vitro* model we have used cannot explore any possible effects that 2′FL may have *in vivo* on the central, sympathetic or parasympathetic nervous systems. In this regard, environmental stress evokes the release of corticotropin-releasing factor (CRF) from the hypothalamus. Yet intestinal dysmotility after stress is dependent on CRF released within the gut while being independent of central CRF secretion [26]. In addition, propulsive PCCs are generated by activity within the enteric nervous system (ENS) and peristalsis as a whole can function independent of connections between the gut and the extrinsic nervous sytem. PCC are completely prevented by the sodium channel blocker tetrodotoxin [16], and we have shown that 2′FL has no direct effect on interstitial cell of Cajal generated circular muscle contractions. Thus, action of 2′FL on the ENS is sufficient explanation for its treatment effect on dysmotility. Additionally, the results from our in vivo faecal pellet expulsion experiments indicate that any effects of 2′FL on the nervous system extrinsic to the gut did not reverse the effects of 2′FL on intestinal propulsive motility.

Intraluminal 2′FL also decreased PCC velocity and frequency in gut colon segments taken from unstressed animals. 2′FL may play a normal role in human health, since it is found in large amounts in normal human breast milk depending on the secretor’s status,. However, other factors in the diet or breast milk could counter the observed effect in healthy individuals serving to maintain homeostasis. Nevertheless, our results support the idea that one possible role of 2′FL is to maintain normal gut motility in the face of environmental stress when homeostatic mechanisms may be insufficient.

Human breast milk contains more than 200 oligosaccharides consisting largely of fucosylated or sialylated molecules [18, 27]. 2′FL is the most commonly abundant HMO in human milk but not in bovine milk where most oligosaccharides are sialylated [18, 28]. The secretion of 2′FL is associated with Lewis blood group secretor status, so that not all mothers possess this HMO in their breast milk. This is an added reason to study its effects in the event of possible translation to the clinical situation. In a previous study, we showed that fucosylated but not sialylated HMO had direct effects on colon motility [15]. Consequently, we would predict that other fucosylated HMO such as 3′FL might have similar dysmotility restoring capability. However, we were not able to acquire sufficient 3′FL to test in our experiments.

HMO, and in particular 2′FL, may have direct effects on the intestinal epithelium but recent evidence suggests that they can be transported across the epithelium into the systemic circulation [29]. Although only a small amount is absorbed (about 0.1% relative to oral dose), they have been associated with certain anti-inflammatory signalling pathways and neuronal development in infants, among other biological effects *in vitro* [29]. Such systemic effects are therefore suggestive of possible direct effects of milk glycans on enteric nerves and muscle.

With respect to 2′FL that is not transported across the epithelium, there is in vitro evidence that HMOs including 2’FL can act directly on intestinal epithelial cell lines to attenuate antigen-antibody complex induced chemokine release [30] or to support gut maturation [31], although there is apparently no similar data using the intact intestine. However, it is well known that intestinal epithelial cells can signal to myenteric intrinsic primary afferent neurons (IPANs). IPANs do not appear to exist in the submucous plexus in the mouse [32, 33]. Thus, the 2'FL would have needed to activate myenteric intrinsic primary afferent neurons (IPANs) to modulate PCC. Myenteric IPANs have processes that extend into the mucosa to ramify next to the epithelium lining the lumen. Consequently, the 2'FL would not have had to diffuse to the myenteric plexus but only to the chemosensory terminals of IPANs which lie adjacent to the luminal epithelium [34].

HMO are generally not digested until they reach the colon where they act as prebiotics and promote the growth of beneficial organisms such as Bifidobacteria. The further effects of fermentation and promotion of bacterial growth in the colon have significant biological local and systemic effects [18, 28]. Since the model system we have used consists of jejunal and colon segments whose contents were flushed out prior to use, and the observed effects occurred within minutes of application, the functional changes we report here in gut motor function are likely to be direct rather than a consequence of alterations in the resident microbiome. This may be especially important since ingested HMO largely reach the large intestine unaltered. Their functions of stimulating the growth of commensal bacteria such as Bifidobacteria offer additional advantages since these also have local immunoregulatory activities including their involvement in fermentation and production of short-chain fatty acids [35]. The latter, individually or together, also have very significant effects on pain perception and the maintenance of epithelial function and integrity [36–39]. Future research should explore in studies like the present one, the effects of oral ingestion of HMO such as 2′FL, and compare results with the effects of other prebiotics.

We have demonstrated, for the first time, that a fucosylated HMO restores restraint stress induced intestinal dysmotility to relative normality for either mouse jejunum or colon segments, in vitro. These results in the murine gut, if clinically translated, would offer potential novel therapeutic opportunities that are testable experimentally.

## Abbreviations

2′FL: 2′-fucosyllactose
Dmaps: Spatiotemporal diameter maps
HMO: human milk oligosaccharide
JB-1: Lactobacillus rhamnosus
PCC: propagating contractile clusters
